# Anxiety and functional impairment affects undergraduate psychology students’ learning in remote emergency teaching during the COVID-19 pandemic

**DOI:** 10.1038/s41598-023-27845-4

**Published:** 2023-01-27

**Authors:** Vitor Rabelo de Sá, Paula Victoria Sozza Silva Gulá, Tamiris Prizon, Raquel Messi Falcoski, Rafael Naime Ruggiero, Fernando E. Padovan-Neto

**Affiliations:** 1 Department of Psychology, Faculty of Philosophy, Sciences and Letters of Ribeirão Preto, University of São Paulo, Ribeirão Preto, São Paulo Brazil; 2 Department of Neurosciences, Faculty of Medicine of Ribeirão Preto, University of São Paulo, Ribeirão Preto, São Paulo Brazil

**Keywords:** Psychology, Anxiety

## Abstract

This study aimed to explore the impact of anxiety and functional impairment measures on a sample of undergraduate psychology students. Learning performance was evaluated during the emergency remote teaching during the first wave and in the post-vaccination period of the COVID-19 pandemic in Brazil. Data modeling revealed that psychometric indicators of severe anxiety and severe functional impairment predicted students with lower learning performance in pairs of pre- and post-test multiple-choice questions. This is the first study to highlight the association between measures of generalized anxiety and functional impairment having a deleterious impact on students’ learning performance. This manuscript highlights that educational policies should be designed to deal with students’ mental health under stressful situations.

## Introduction

The coronavirus disease 2019 (COVID-19) pandemic affected education worldwide. Several institutions adopted remote teaching to reduce the spread of the coronavirus^1^. The consequences of suspending in-person activities in education losses are still unknown. A global survey concerning the impact of COVID-19 on higher education^2^ reported that institutions were unprepared to engage and motivate students during emergency distance teaching and learning. The COVID-19 pandemic also increased the number of cases of mental illness around the globe. Specifically, a significant proportion of the world’s population has experienced symptoms of anxiety^3,4^, including coronavirus anxiety^5,6^. Brazil has the world’s highest prevalence of anxiety^7^, and the COVID-19 pandemic has generated a social, economic, and sanitary crisis that either increased or aggravated anxiety levels in the population^8^. Groups with severe anxiety symptoms are frequent in higher education institutions^9,10^. Segments that have been particularly affected by the pandemic are women and young adults^11^.

Psychological distress is an important mental health problem among higher education students^12,13^. Unfortunately, the COVID-19 pandemic incorporated new stressors into an already stressful routine of undergraduate students. Following online teaching and maintaining a virtual environment that provides adequate conditions for optimal monitoring can be stressful, especially in a high level of uncertainty such as the COVID-19 pandemic. The interruption of face-to-face classes, abrupt schedule changes, adaptation to virtual environments, social distancing, and insecurity regarding the teaching–learning process can trigger or intensify anxiety^14,15^.

The deleterious impacts of stressful events on students’ academic performance have been widely discussed in the literature^16,17^. Physiological and subjective measures of anxiety are directly linked to learning and memory^18,19^. Severe anxiety can disrupt students’ capacity to receive, process, and retrieve information, leading to poor learning capacity and impacting academic achievement^20,21^. Anxiety can also disrupt essential processes involved in learning, such as concentration and working memory^22^. Understanding the effects of COVID-19-induced anxiety on learning performance is crucial to improving academic achievement.

During the COVID-19 pandemic, students experienced impaired functioning in their studies, home management, social and private leisure activities, and social relationships^23^. Functional impairment can aggravate or trigger anxiety symptoms^24^. Although it has been demonstrated that students with excessive anxiety are more likely to experience learning difficulties, the association between anxiety and impaired functioning on students’ learning performance has yet to be investigated. Based on this research gap, this study’s main goal was to determine whether severe functional impairment and anxiety affect students’ performance in remote education during the COVID-19 pandemic. We hypothesized that high anxiety and excessive functional impairment would contribute to lower learning performance among undergraduate students engaged in remote learning courses. Therefore, this study tracked the performance of undergraduate psychology students in pairs of pre- and post-classes multiple-choice questions during a one-semester psychopharmacology course that occurred during the first wave and in the post-vaccination period of the COVID-19 pandemic in Brazil. At the end of the semester, measures of functional impairment and anxiety were compared with students’ academic performance.

## Methods

### Participants and procedure

#### Study design

This study collected data from Brazilian adults to evaluate the performance of undergraduate psychology students. Pairs of pre- and post-classes multiple-choice questions were collected during a one-semester psychopharmacology course that occurred during the first wave and the post-vaccination period of the COVID-19 pandemic in Brazil. At the end of the semester, functional impairment and anxiety measures were compared to academic performance. Psychometric scores taken from the undergraduate psychology students’ sample were compared to those of a random sample of undergraduates and the general population. In this study, we first provide an overview of general anxiety, functional impairment, and coronavirus anxiety in the years 2020 (i.e., first wave) and 2021 (i.e., post-vaccination) in samples of psychology undergraduates, students with incomplete higher education, and the general population. Second, we investigated the relationship between generalized anxiety and measures of functional impairment and the performance of psychology undergraduates during emergency remote teaching. Finally, we examined whether the association between generalized anxiety and functional impairment measures could account for the relationship between psychological state and learning among psychology undergraduates.

#### General population participants recruited in an online survey

A nonprobabilistic snowball sampling technique was used to collect data with online surveys. Participants were recruited using social media platforms (e.g., Facebook, WhatsApp, and Instagram) and were asked to volunteer for this research project without compensation. Before providing informed consent, the participants were given details regarding the nature, objectives, risks/benefits, and anonymity of responses for this study. The first online survey collected data from 518 Brazilian adults during the first wave of the COVID-19 pandemic between July 31 and September 13, 2020^11^. During this period, the 7-day rolling average case fatality rate due to COVID-19 infection was relatively stable, with an average of 2.02% (from 1.76 to 2.31%)^25^. This period was characterized by strict protective measures to ensure prevention against COVID-19 (e.g., physical distancing, use of protective equipment, thermal screening, and frequent sanitization). Cases increased from 2.67 million to 4.33 million, and deaths rose from 92,728 to 131,746. The second online survey collected data from 427 Brazilian adults in the post-vaccination period between October 25, 2021 and February 8, 2022. Throughout this time, the 7-day rolling average case fatality rate due to COVID-19 infection reduced from 3.14% in October 2021 to 0.43% in February 2022^25^. Protective measures such as physical distancing, use of protective equipment, thermal screening, and frequent sanitization were still mandatory. During this period, the number of cases increased from 21.74 million to 26.79 million, and the number of deaths rose from 606,114 to 634,118. Further, there was an increment from 52.13 to 70.74% of people who completed the vaccination protocol^25^. The sociodemographic characteristics (i.e., age, sex, region, exposure to someone with COVID-19, previous diagnosis of COVID-19, friend or family member that died from COVID-19, vaccination status, and previous diagnosis of anxiety disorder) are indicated in Supplementary Table 1.

The sample size was not formally an objective for the general population sample recruited in the online survey since we tried to obtain the maximum number of participants in each period. However, the minimum sample required to detect a weak effect size (0.2) was 64 samples for a power of 80% using a two-sided significance level of 5% for the unpaired t-test. The achieved post-hoc statistical power to detect WSAS (Work and Social Adjustment Scale), CAS-BR (Coronavirus Anxiety Scale), and GAD-7 (Generalized Anxiety Disorder Scale; not significant) scores using the sample of 2020 and 2021 is 1.00, 0.99, and 0.23, respectively.

#### Undergraduate psychology students recruited in an online survey

A nonprobabilistic convenience sampling was used to collect data from undergraduate students. A total of 51 psychology undergraduate students of the Faculty of Philosophy, Sciences, and Letters of Ribeirão Preto, from the University of São Paulo, participated in this study. Before providing informed consent, participants were informed about the nature, objectives, risks/benefits, and anonymity of responses for this study. Inclusion criteria were age 18 and voluntary participation in the survey without compensation. The remote psychopharmacology courses occurred during the first wave of the COVID-19 pandemic (between October 5 and December 2020, N = 27) and during the post-vaccination period (between August 16 and December 20, 2021, N = 24). During the period covering the first course in 2020, the 7-day rolling average case fatality rate due to COVID-19 infection was relatively stable, with an average of 1.97% (ranging from 1.38 to 3.44%)^25^. During the period covering the second course in 2021, the 7-day rolling average case fatality rate due to COVID-19 infection was relatively stable, with an average of 2.38% (ranging from 1.45 to 3.56%)^25^. In both periods, protective measures were the same, as indicated above. The sociodemographic characteristics (i.e., age, sex, region, vaccination status, and previous diagnosis of anxiety disorder) are indicated in Supplementary Table 1.

In this sample, we aimed for a minimum of 15 participants, which was the sample size to detect the expected differences in pre- and post-classes multiple-choice questions based on our previous publication^23^. This sample size was calculated for a power of 80%, an expected effect size of 0.8 a two-sided level of significance of 5% for the paired t-test. The achieved post-hoc statistical power with the total sample of 51 (pre vs. post) was 1.00.

### Measures

#### Coronavirus anxiety

The Brazilian adapted version of the Coronavirus Anxiety Scale (CAS-BR) is a psychometric instrument used to measure coronavirus-related fear and anxiety^5,11^. The CAS-BR items measure physiologically-based symptoms experienced when triggered by coronavirus-related information and thoughts. Using a 5-point time-anchored scale (0 = not at all to 4 = nearly every day over the last 2 weeks), participants rated how frequently they experienced each anxiety symptom. Lee’s replication of the coronavirus anxiety study proposed a CAS cut-off score of ≥ 5 when assessing the general population^26^. Although the Brazilian study did not identify the optimal cut-score for psychiatric screening purposes^11^, we used a CAS-BR cut-off score of ≥ 5 for measuring coronavirus anxiety in our study sample. The CAS-BR exhibited internal consistency reliability (Cronbach’s α) ranging from 0.76 to 0.88 in the study samples (Supplementary Table 1).

#### Generalized anxiety

The adapted version of the Generalized Anxiety Disorder (GAD-7) scale is often used to indicate clinical symptoms of generalized anxiety^27^. Students were asked to rate seven items of the GAD-7, using a 4-point time-anchored scale (0 = not at all, 1 = several days, 2 = more than half of the days, and 3 = nearly every day), regarding how frequently they experienced symptoms of generalized anxiety over the past 2 weeks. GAD-7 score is given by the sum of the points of the 7 questions, varying between 0 and 21 points. According to the original manuscript^27^, cut points of 5, 10, and 15 represented mild, moderate, and severe anxiety levels. The Brazilian adaptation of the GAD-7^26^ presented an excellent indicator regarding validity and reliability^28^ . The GAD-7 exhibited internal consistency reliability (Cronbach’s α) ranging from 0.82 to 0.93 in the study samples (Supplementary Table 1).

#### Functional impairment

The adapted version of the Work and Social Adjustment Scale (WSAS) is a psychometric instrument used to measure functional impairment^11,29^. The WSAS is a five-item scale that measures (1) the ability to work or study; (2) home management; (3) social leisure activities; (4) private leisure activities; and (5) the ability to form and maintain close relationships with others. A WSAS score above or equal to 21 suggests moderate to severe psychopathology; scores between 10 and 20 are related to significant functional impairment, and scores below 10 appear related to subclinical symptomatology. The WSAS^30^ scale was designed to measure functional impairments resulting from a given problem (e.g., fear and anxiety over the coronavirus). It has been used with symptoms-based instruments (such as the GAD-7) to track social functioning in patients with anxiety and depression^31^. According to previous studies^11,29^, there is good convergent validity between GAD-7 and WSAS. Interestingly, it has been suggested that WSAS and GAD-7 assess slightly different aspects of psychological distress^32^. Therefore, WSAS is a valuable screening tool with the potential to provide additional outcome measures. The WSAS exhibited internal consistency reliability (Cronbach’s α) ranging from 0.67 to 0.86 in the study samples (Supplementary Table 1).

#### Measures of learning

Measures of learning were obtained quantitatively using pre- and post-test multiple-choice questions within a virtual learning environment as described elsewhere^23^. We developed a series of multiple-choice questions distributed across seven study modules allocated in a one-semester psychopharmacology course. All questions encouraged critical thinking about psychopharmacology and knowledge relevant to clinical psychology practice. Sets of study materials were selected for each module and made available to students at the University of São Paulo online platform one week before the online lectures. Students answered the same multiple-choice questions at an online platform (Nearpod) before (pre-test) and after (post-test) a 90-min lecture. At the end of the course, students were asked to complete an online survey on Google Forms platform to report demographic information, coronavirus anxiety, generalized anxiety, and functional impairment.

Only students who had completed all pre- and post-tests for a given study module were included in this study. Incomplete answers (i.e., students that responded only to the pre- or the post-test) were excluded from the analysis. Also, a few students did not participate in all study modules. Therefore, not all students answered the same number of pairs of questions during this study. Considering all participating students and excluding incomplete answers, 1636 pre- and post-test pairs were computed across the seven study modules. There was a total of four possible combinations for the pairs multiple-choice questions: correct-correct (CC; 909 pairs), incorrect-correct (IC; 386 pairs), correct-incorrect (CI; 102 pairs), and incorrect-incorrect (II; 239 pairs). Finally, the data were normalized to the range of 0 and 100%, considering each student’s performance in the course (i.e., the normalized sum of all four combinations of answers to pairs of multiple-choice questions in all seven study modules). Therefore, if a student answered a question incorrectly on the first attempt (pre-test), he would have a chance to aim for the correct answer on the second attempt (post-test). The combination of incorrect to correct answers was used to measure learning since it indicates that students could learn new content following a 90-min lecture.

### Statistical analysis

The demographic and psychometric characteristics of the sample were summarized as mean (M) and standard deviation (SD). Continuous data normality was checked out with the Shapiro–Wilk. Additionally, we inspected the normality of the residuals for every model used, using the QQ plot and the residual against fitted values to verify the linear model assumptions. Comparisons between groups were performed using the unpaired Student’s t-test and two-way ANOVA for repeated measures. Pairs of answers to multiple-choice questions were treated as categorical data (expressed as proportions) and analyzed using the Chi-square and Fisher’s exact test. Pairs of multiple-choice questions were normalized to the maximum number of questions each student answered in all four study modules for correlations with CAS-BR, GAD-7, and WSAS scores. Pearson’s correlation coefficient assessed the correlation between continuous variables (i.e., (i) psychometric instruments: GAD.SUM, WSAS.SUM, GAD > 15, and WSAS ≥ 21; (ii) answers to pairs of multiple-choice questions: correct-correct (CC), incorrect-correct (IC), correct-incorrect (CI), and incorrect-incorrect (II); the proportion of answers in the pre- and post-tests; and (iv) the number of Participation in the multiple-choice test). To understand multivariable associations, we used multiple linear regression with no penalization. We reported the effect size using Cohen’s d (d) for numerical variables. We reported the odds ratio (OR) and Cramer’s V (V) for categorical variables. All analyses were performed using Graphpad Prism and R software. Cohen’s d (d) and Cramer’s V (V) were calculated online using Statistics Kingdom (https://www.statskingdom.com/effect-size-calculator.html). Data was presented by the mean ± standard error of the mean (SEM). The significant *p*-value was set to 0.05.

To identify clusters of data that could explain the relationship between learning and mental status, we performed a non-supervised approach using the Gaussian mixture model (GMM). The GMM is a probabilistic model that assumes the data combines independent Gaussian distributions estimating the mean and variance of an optimal number of Gaussians. We used the MClust algorithm in R, which performs a model-based clustering based on parameterized finite Gaussian mixture models. The optimal model is selected according to Bayesian Information Criterion—BIC^33^.

### Ethics

The study was conducted according to the guidelines of the Declaration of Helsinki. All procedures performed in this study were approved by the ethics committee of the Faculty of Philosophy, Sciences, and Letters of Ribeirão Preto at the University of São Paulo (FFCLRP/USP; CAAE 32077620.1.0000.5407 and CAAE 51322521.3.0000.5407). Written informed consent was obtained from all subjects involved in the study.

## Results

### Measures of generalized anxiety, functional impairment, and coronavirus anxiety during the first wave (late 2020) and in the post-vaccination period (late 2021) of the COVID-19 pandemic

**Figure 1:**
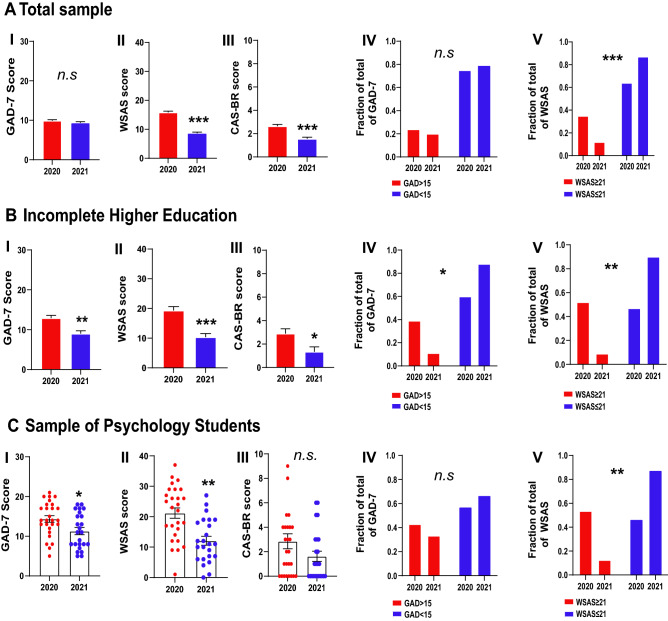
Comparisons of levels of anxiety, functional impairment, and coronavirus anxiety. **(A)** The total sample comprises the general population (N = 518 in 2020 and N = 427 in 2021). **(B)** Incomplete Higher Education group (N = 53 in 2020, and N = 42 in 2021) and **(C)** Psychology students’ group (N = 27 in 2020, and N = 24 in 2021). Comparison of 2020 and 2021 scores of (**I**) GAD-7, (**II**) WSAS, and (**III**) CAS-BR. Comparison of the proportion of (**IV**) GAD-7 > 15 and the (**V**) WSAS ≥ 21 scores between 2020 and 2021. The values were expressed by means ± SEM, the p-value was indicated by *p < 0.01, **p < 0.001, and ***p < 0.0001 and ‘n.s.’ indicating no difference among the samples. *GAD-7* Generalized anxiety disorder scale, *WSAS* Work and social adjustment scale, *CAS-BR* Brazilian adapted version of the coronavirus anxiety scale.

We first evaluated measures of generalized anxiety, functional impairment, and coronavirus anxiety in the years 2020 and 2021 in two samples taken from the general population (945 participants/N = 518 in 2020 and N = 427 in 2021), individuals with incomplete higher education (95 participants/N = 53 in 2020 and N = 42 in 2021), and psychology undergraduate students (51 participants/N = 27 in 2020 and N = 24 in 2021) (Fig. 1).

Group comparisons (unpaired Student’s t-test) in the total sample revealed that GAD-7 scores did not change in 2021 (t(943) = 1.34, p = 0.1793, d = 0.08, Fig. 1A-I). WSAS (t(943) = 11.55, p < 0.0001, d = 0.76, Fig. 1A-II) and CAS-BR (t(943) = 5.39; p < 0.0001, d = 0.35, Fig. 1A-III) scores reduced in 2021. The proportion of individuals from the general population with GAD-7 scores above the cut-off point (> 15) was similar in 2020 (N = 128/24.7%) and 2021 (N = 88/20.6%, Fisher’s exact test, p = 0.1399, OR = 1.26, Fig. 1A-IV). The proportion of individuals in the general population with WSAS scores above the cut-off point (≥ 21) reduced from 2020 (N = 183/35.3%) to 2021 (N = 52/12.2%, Fisher’s exact test, p < 0.0001, OR = 3.96, Fig. 1A-V).

Interestingly, group comparisons (unpaired Student’s t-test) in individuals with incomplete higher education revealed a significant reduction in GAD-7 (t(93) = 3.06, p = 0.0028, d = 0.75, Fig. 1B-I), WSAS (t(93) = 4.82, p < 0.0001, Fig. 1B-II), and CAS-BR scores (t(93) = 2.59, p = 0.0109, d = 0.54, Fig. 1B-III). The proportion of individuals with incomplete higher education with GAD-7 scores above the cut-off point (> 15) was similar in 2020 (N = 21/39.6%) and 2021 (N = 5/11.9%, Fisher’s exact test, p = 0.0028, OR = 0.00, Fig. 1C-IV). The proportion of psychology students with WSAS scores above the cut-off point (≥ 21) was reduced from 2020 (N = 27/50.9%) to 2021 (N = 5/11.9%, Fisher’s exact test, p < 0.0001, OR = 7.70, Fig. 1C-V).

The results found in the group of psychology students followed the same pattern as the sample of individuals with incomplete higher education. Group comparisons (unpaired Student’s t-test) revealed a reduction in GAD-7 scores (t(49) = 2.50; p < 0.015, d = 0.70, Fig. 1C-I), WSAS scores (t(49) = 3.98, p = 0.0002, d = 1.12, Fig. 1C-II), and no change in CAS-BR scores (t(49) = 1.61; p = 0.1136, d = 0.45, Fig. 1C-I). The proportion of psychology students with GAD-7 scores above the cut-off point (> 15) was similar in 2020 (N = 12/42.9%) and 2021 (GAD > 15: N = 8/33.4%, Fisher’s exact test, p = 0.5729, OR = 1.50, Fig. 1C-IV). The proportion of psychology students with WSAS scores above the cut-off point (≥ 21) was reduced from 2020 (N = 15/53.6%) to 2021 (N = 3/12.5%, Fisher’s exact test, p = 0.0030, OR = 8.07, Fig. 1C-V).

### Relationship between learning performance, generalized anxiety, and functional impairment

**Figure 2:**
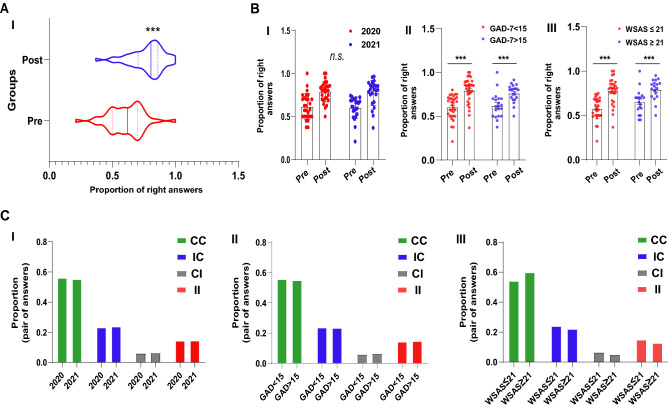
Assessment of students’ psychological state and learning performance during emergency remote teaching. **(A-I)** Comparison between pre- and post-test averages. **(B-I)** Comparison of performance in pre- and post-test of individuals between 2020 and 2021; **(II)** GAD-7 > 15 or < 15, and **(III)** WSAS ≥ 21 or ≤ 21. **(C-I)** Comparison of the proportion of categorical answers between **(I)** 2020 and 2021, **(II)** GAD-7 > 15 or GAD-7 < 15, and **(III)** WSAS ≥ 21 or ≤ 21. The results were presented by means ± SEM, and the p-value was indicated by *p < 0.01, **p < 0.001, and ***p < 0.0001 and ‘n.s.’ indicated no difference among the samples. Answers to pairs of multiple-choice questions are indicated as correct–correct (CC), incorrect–correct (IC), correct–incorrect (CI), and incorrect–incorrect. *GAD-7* Generalized anxiety disorder scale, *WSAS* Work and social adjustment scale.

We used pre- and post-test multiple-choice questions to obtain a quantitative measure of learning in psychology students during emergency remote education. Our data showed a significant increase in the number of correct answers to multiple-choice questions in the post-test, confirming the potential utility of pre- and post-test multiple-choice questions as a measure of learning (unpaired Student’s t-test, t(50) = 8.73, p < 0.0001, d = 1.28, Fig. 2A-I).

The impact of generalized anxiety and functional impairment was investigated in the learning performance of two samples of psychology students. Two-way repeated-measures ANOVA revealed that students’ performance did not differ between the first wave and the post-vaccination period of the COVID-19 pandemic (F(1,49) = 0.15, p = 0.6913, d = 0.01, Fig. 2B-I). When pooling the data from both years, the analysis revealed that students with GAD-7 (F(1,49) = 0.14, p = 0.7054, d = 0.05, Fig. 2B-II) and WSAS (F(1,49) = 1.61, p = 0.2393, d = 0.37, Fig. 2B-III) scores below or above the cut-off points had similar performance in pairs of multiple-choice questions. However, the rate of correct answers was higher in post-class when compared to the rate of the pre-class, regardless of the year (F(1, 49) = 75.17, p < 0.0001, Fig. 2B-I), the score obtained in GAD-7 (F(1,49) = 73.56, p < 0.0001, Fig. 2B-II) and WSAS score (F(1,49) = 65.11, p < 0.0001, Fig. 2B-III).

Finally, we investigated if generalized anxiety and functional impairment measures would impact students’ answers to pairs of pre- and post-tests (Fig. 2C). The Chi-Square test revealed that the proportion of responses in both years was similar (X^2^(3) = 0.10, p = 0.9907, V = 0.01, Fig. 1C-I) and independent of generalized anxiety levels (X^2^(3) = 0.24, p = 0.9692, V = 0.01, Fig. 2C-II) or functional impairment (X^2^(3) = 4.20, p = 0.2399, V = 0.05, Fig. 2C-III).

### Prediction of performance in the teaching–learning process through data modeling

**Figure 3:**
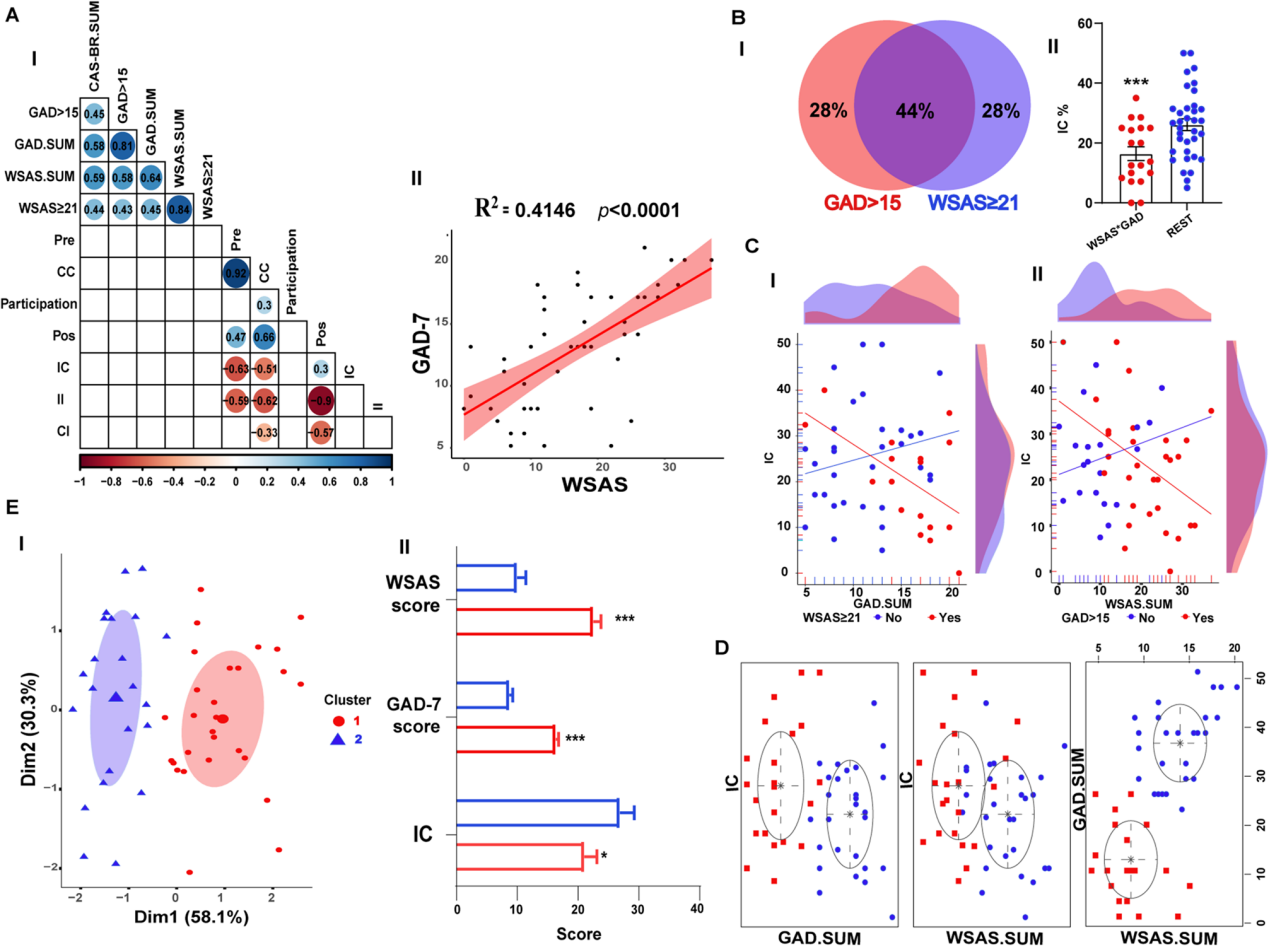
Data modeling indicates that high levels of anxiety and dysfunctionality negatively impact students’ learning performance. **(A-I)** Correlation matrix of (i) the scores in the psychological tests (GAD.SUM, WSAS.SUM, GAD.15, WSAS.21, and CASBR), (ii) answer categories (CC, IC, CI, and II), (iii) the proportion of answers in the pre and post-test (Pre and Pos), and (iv) Sex. **(II)** Positive correlation between GAD-7 and WSAS. **(B-I)** The Venn Diagram represents the intersection between individuals with scores higher than 15 in GAD-7 and greater than or equal 21 in the WSAS test. **(II)** Comparison between the individuals with higher scores of GAD-7 and WSAS and the percentage of IC category. **(C-I)** A Bimodal distribution is observed by analyzing the GAD-7 score by cut-off from WSAS ( ≥ 21) and **(II)** the WSAS score by cut-off from GAD (> 15). **(D)** The Gaussian mixture model indicates that data comes from two Gaussian diagonal distributions. **(E-I)** Representation of GAD, IC, and WSAS distribution values in the two theoretical clusters, and **(II)** the different profiles of psychological status and learning process in clusters 1 and 2. To indicate no significative differences between the groups we use ‘n.s.’, and the significant differences were listed as *p < 0.01, **p < 0.001, and ***p < 0.0001. Answers to pairs of multiple-choice questions are indicated as correct–correct (CC), incorrect–correct (IC), correct–incorrect (CI), and incorrect–incorrect. *GAD-7* generalized anxiety disorder scale, *WSAS* Work and social adjustment scale.

We also performed bivariate and multivariate analyses to explain the association between psychological state (i.e., generalized anxiety and functional impairment) and learning performance (i.e., changes from an incorrect to a correct answer to pairs of pre- and post-tests). As expected, answer categories and testing period correlated with each other such as CC category and the pre-test (r = 0.92), II category and the post-test (r =  − 0.9), and IC with pre-test (r =  − 0.63) (Fig. 3A-I).

The correlation clusters indicated a positive interaction between GAD.SUM e WSAS.SUM (r = 0.64), also evidenced by linear regression analysis indicating a coefficient of R^2^ = 0.4146 (p < 0.0001) (Fig. 3A-II). We also had the WSAS.SUM with WSAS ≥ 21 (r = 0.84), and GAD.SUM with GAD > 15 (r = 0.81). We also built multivariate linear regression models to identify associations between WSAS, GAD-7, and learning performance. We first identified the interaction of WSAS ≥ 21 and GAD-7 > 15 as a significant predictor for the number of responses of the type IC (adjusted R^2^ = 0.10, F(3,47) = 2.97, and p = 0.041, Table 1). This interaction is significant even controlling for sex as a variable (Model 2, adjusted R^2^ = 0.11, F(4,46) = 2.661, and p = 0.044, Table 1) and, more importantly, for year (Model 3, adjusted R^2^ = 0.10, F(5,45) = 2.18, and p = 0.07, Table 1). These data suggest that GAD > 15*WSAS ≥ 21 interaction negatively impacts learning performance.

**Table 1:** Results of linear regression models for students with high levels of anxiety and functional impairment as dependent variables.

Variables	B	SE(B)	t	*p* value
Model 1				
Intercept	23.378	2.570	9.096	< 0.0001***
WSAS ≥ 21	12.838	8.127	1.580	0.121
GAD > 15	4.66	3.812	1.222	0.228
WSAS ≥ 21*GAD > 15	− 22.552	9.023	− 2.499	0.016*
Model 2				
Intercept	22.607	2.625	8.613	< 0.0001***
WSAS ≥ 21	13.609	8.098	1.681	0.0996
GAD > 15	3.889	3.836	1.014	0.3160
Male	4.629	3.645	1.27	0.2105
WSAS ≥ 21*GAD > 15	− 22.938	8.97	− 2.557	0.0139*
Model 3				
Intercept	24.517	4.182	5.863	< 0.0001***
WSAS ≥ 21	12.82	8.265	1.551	0.1279
GAD > 15	2.96	4.172	0.709	0.4818
Male	4.376	3.696	1.184	0.2427
Year	− 2.242	3.802	− 0.59	0.5587
WSAS ≥ 21*GAD > 15	− 22.786	9.038	− 2.521	0.0153*

WSAS ≥ 21*GAD > 15: interaction between WSAS ≥ 21 scores and GAD > 15 scores.

Statistics: Estimated (B), Standard Error (SE), t value (t) and *p* value (*p*) are shown across the table (0; ‘***’0.001; ‘**’0.01; ‘*’0.05; ‘0.1’; ‘1’).

Model 1: R^2^ = 0.1594; adjusted R^2^: 0.10.

Model 2: R^2^ = 0.1879; adjusted R^2^: 0.11.

Model 3: R^2^ = 0.1941; adjusted R^2^: 0.10.

Given the indications of our regression models, we decided to check what percentage of subjects had a higher score than WSAS ≥ 21 and GAD > 15. The Venn diagram indicated that 44% of the sample from GAD>15 or WSAS ≥ 21 (21% of the total sample) shared this profile (Fig. 3B-I). This same portion showed a lower learning performance than the remaining participants (unpaired Student’s t-test, t(52) = 3.048; p = 0.0036, d = 0.05, (Fig. 3B-II).

Furthermore, when we examined the GAD-7 score by cut-off from WSAS (≥ 21) (Fig. 3C-I) or the WSAS score by cut-off from GAD-7 (> 15) (Fig. 3C-II), we observed a bimodal distribution in the interaction between the increasing GAD-7 score with a lower rate of correct answers in the post-test, as well as in individuals with a score greater than 20 in the WSAS, thus suggesting that two Gaussian distributions can generate the data.

In this sense, we applied a Gaussian mixture model algorithm to identify the optimal distribution of the data. We found that the optimal representation of the data is using 2 diagonal clusters (equal volume and equal shape, log-likelihood =  − 514.1697, n = 51, df = 10, BIC =  − 1067.658) (Fig. 3D). These theoretical clusters reflect different learning and psychological status profiles. Cluster 1 was ranked by higher scores on the WSAS (GMM-means = 22.52), and on the GAD-7 (GMM-means = 16.34) and higher scores on the IC category (GMM-means = 21.05), differing from cluster 2, which encompasses lower scores on the WSAS (GMM-means = 9.99), on the GAD-7 (GMM-means = 8.72) and better performance for IC category (GMM-means = 26.88) (Fig. 3E-I), with a significant difference between the groups using Two-way ANOVA (difference between clusters: F(1, 147) = 14.73, p = 0.002), with Sidak’s multiple comparison test indicating differences for WSAS (p < 0.0001, d = 0.53), GAD-7 (p = 0.0016, d = 1.86) and IC (p = 0.022, d = 3.00).

## Discussion

This study aimed to explore the impact of anxiety and functional impairment measures on a sample of undergraduate psychology students’ learning performance during the emergency remote teaching in a psychopharmacology course conducted during the first wave and post-vaccination period of the COVID-19 pandemic in Brazil. Data revealed that students learned new content during the emergency remote learning with similar performance in classes conducted in the two periods of the COVID-19 pandemic. Strikingly, students with severe anxiety and functional impairment had lower performance in pairs of the same multiple-choice questions answered before (pre-test) and after (post-test) a 90-min lecture (i.e., were less likely to change from an incorrect to a correct answer). Our study highlights that the association between measures of generalized anxiety and functional impairment has a deleterious impact on students’ learning performance.

Students engaged in higher education are more likely to be exposed to additional stressors. A systematic quantitative review revealed an overall prevalence of anxiety symptoms in 41% of university students (33% in Asia, 51% in Europe, and 56% in the US) during the COVID-19 pandemic^34^. Factors arising from the COVID-19 pandemic such as financial difficulty, less social interaction, poor sleep quality, fear of infection, lack of physical activity, and loneliness increased anxiety among undergraduate students^35^. A study conducted during Brazil’s first wave of the COVID-19 pandemic (September to October 2020) revealed that 52.5% of undergraduate students had anxiety symptoms^36^. In agreement with this study, our sample revealed that 39.6% of individuals with incomplete higher education and 42.9% of psychology undergraduate students presented high anxiety levels during the first wave of the COVID-19 pandemic. Further, our data revealed that high anxiety levels reduced to 11.9% in the sample of individuals with incomplete higher education, and to 33.4% in the sample of psychology undergraduate students in the post-vaccination period. A possible explanation for the reduction of anxiety levels among undergraduate students may be related to the widespread vaccination of the population for COVID-19. Although studies conducted in Brazil^8^ and other countries^37,38^ reported a reduction in measures of mental health distress after COVID-19 vaccination, levels are still higher than in the pre-pandemic period.

Studies have demonstrated that anxiety impairs learning ability^39,40^. The difference between the learner’s performance before (pre-test) and after (post-test) the teacher’s intervention is a valuable measure of learning^41,42^. Therefore, changes from incorrect to correct in pairs of multiple-choice questions are likely to indicate that students could learn new content during the class. Using this measure as a learning indicator, we demonstrated, for the first time, that students who presented exacerbated symptoms of generalized anxiety and functional impairment had lower performance in pairs of multiple-choice questions. This data indicates that the combination of high levels of anxiety and functional impairment may be associated with students’ learning difficulties.

Even under stressful situations, students’ positive performance indicates the importance of effective teaching strategies^43–45^. Educators who employ an autocratic and teacher-centered approach in the classroom are more likely to elicit higher anxiety levels in students^46^. On the other hand, students who learn under a democratic and student-centered approach, such as the methodology used in this study, display better academic achievements^47^. Active methodologies (such as the flipped classroom) mediated by Information and Communication Technologies (ICTs) can overcome dysfunctional and anxiogenic states to favor the learning process^48,49^. The high percentage of pairs of correct answers detected in both pre- and post-tests in this study (56.4% in 2020 and 55.2% in 2021) suggests that students were engaged in active learning (i.e., they consulted the material available online one week before the classes). Unfortunately, using ICTs alone does not guarantee the necessary involvement of the student for meaningful learning. Culture, infrastructure, digital equity, and digital privacy were critical factors for online learning during COVID-19^50,51^. In this sense, a large study conducted in China revealed that the lack of course support (i.e., arrangement, assessment, and learning) and academic support (i.e., interaction with tutors and schoolmates) had a deleterious effect on both students’ performance and mental health^52^.

Taken together, the results of this study have educational and clinical implications*.* The COVID-19 pandemic has caused a substantial disruption in the education system in higher education institutions worldwide^53^ and caused a negative impact on students’ quality of life^54,55^. In Brazil, factors that correlated with low quality of life are being young, being female, having a high frequency of self-report for anxiety and depression, having high levels of burnout, and having a worse perception of online learning^11,56^. Considering the stressful life events caused by the COVID-19 pandemic in educational settings, the results presented here reinforce the need for new strategies to deal with stress consequences. Although the recent literature demonstrates that the COVID-19 pandemic contributed to poor academic performance among higher education students, many educators do not understand how psychological distress can affect them. Our study highlights that educators should be aware of the impact of anxiety and functional impairment on students’ learning achievements. Further, educators should be able to recognize what classroom conditions make the students anxious and determine better learning and teaching environments^57^. As functional impairment and anxiety may exist in a common framework, targeting both concurrently in a clinical context may improve students’ mental health and lead to better academic achievements. In this sense, effective coping strategies can be potentially protective under stressful situations. Previous reports have indicated that students who are more flexible in their coping strategies are less vulnerable to psychological distress compared to less flexible students^58–60^. Therefore, a better understanding of students’ coping strategies may guide students in facing the psychological challenges related to the academic routine.

It is essential to highlight the limitations of the study. The two major constraints in our work are the sampling bias and potential confounding factors that were not measured in the current methodology. The sampling method was not probabilistic, so we cannot claim that they represent the entire Brazilian population. Furthermore, since this study was conducted at the University of São Paulo, the differences in students’ demographic characteristics will likely affect the results when applied to other universities. To note, the psychology course curriculum in Brazil follows the National Curriculum Guidelines. It must include the following axes: epistemological and historical foundations, basic psychological processes and phenomena, methodological foundations, procedures for scientific investigation and professional practice, and interfaces with related fields of psychological knowledge and professional practices. Despite the apparent limitations, the sampling methods were chosen since they significantly reduced the cost, the complexity, and, more importantly, the time to complete the study. It is crucial to emphasize that these were fundamental characteristics of this study given the COVID-19 pandemic context. The cross-sectional method used in this manuscript limits the interpretation of the GAD-7 and WSAS measures. No available data was collected before the COVID-19 pandemic to compare psychometric and academic performance indicators. We also evaluated a small sample of undergraduate students in a particular context of remote learning, and we could not map differences across gender and ages. Despite these limitations, our aim using the general population data was to investigate if the same trend observed in the small sample of higher education students (i.e., reduction of GAD-7 and WSAS scores) was observed in a larger population. The other major limitation concerns variables that could affect learning but were not measured in the study. Constructs such as intelligence, self-efficacy, attention, and motivation, or potential problems in delivering the learning content could affect the learning performance and provide helpful information that may underlie psychological distress and learning difficulties. Despite the potential interaction of anxiety and functional impairment affecting academic performance, the low variance explainability of the regression models (i.e., the model explained 10% of the variability) is likely to reflect the lack of measures of other constructs.

Despite the limitations, this study has several strengths. The study is essential since it measures psychometric and academic performance indicators during the pandemic. Our view is that despite the limitations described, the approach provides essential suggestions for understanding how a complex and highly dynamic situation impacts mental health and learning. Furthermore, the sample of higher-education students itself is precious. Students’ learning performance was evaluated during remote emergency teaching during the pandemic period in 2020 and 2021. In this sense, we highlight the advantage of this approach over an artificial experimental environment that does not adequately simulate the learning conditions intended to be studied. Although other studies have demonstrated a relationship between anxiety and academic performance during the COVID-19 pandemic, this is the first study to indicate that the interaction between generalized anxiety and functional anxiety impairment measures in learning performance impairs students’ learning.

## Conclusion

Our results point to crucial constructs that should be included and investigated in future research conducted in the academic environment, such as functional impairment and anxiety. The data presented here further advance our understanding of the relationship between mental health and remote education. Our findings fill a gap in the literature, showing that high levels of anxiety and functional impairment have a negative impact on learning performance when they interact. Future studies should investigate whether there is a relationship between anxiety and functional impairment in academic performance during a classroom-based course.

The results of this study can immediately lead to improved guidance for all working in the educational system. The assessment criteria described in this manuscript can gauge the student’s success in a particular course. This kind of evaluation may help instructors, tutors, and academic leaders substantially with improved lesson preparation, and the adoption of strategies and methodologies more successful in each environment. A potential tool for students and educators is a better understanding of the connection between mental health and academic achievement. Finally, based on the current study’s findings, our observations can assist governments in developing public policies.

## Supplementary Information

Supplementary Table 1.

## Data Availability

The datasets used and analyzed during the current study are available from the corresponding authors upon reasonable request.
