# Supplementary material for: Anxiety and functional impairment affects undergraduate psychology students’ learning in remote emergency teaching during the COVID-19 pandemic
Source: Sci Rep. 2023 Jan 27;13:1503. doi: 10.1038/s41598-023-27845-4 (PMC9880938; doi:10.1038/s41598-023-27845-4)
Supplement: Supplementary file 1 — Supplementary Table 1. [file 41598_2023_27845_MOESM1_ESM.docx]

**Supplementary Table 1.** Sociodemographic and psychometric data obtained from all participants.

|  | **General Population** | | **Incomplete Higher Education** | | **Undergraduate Psychology Students** | |
| --- | --- | --- | --- | --- | --- | --- |
|  | **2020** | **2021** | **2020** | **2021** | **2020** | **2021** |
| ***Age (Mean ± SD)*** | 35.45 ± 13.29 | 33.96 ± 13.36 | 21.94 ± 2.82 | 22.62 ± 4.34 | 21.22 ± 1.74 | 21.75 ± 2.67 |
| ***Sex (%)*** |  |  |  |  |  |  |
| Male | 60.23 | 72.37 | 41.51 | 26.19 | 29.63 | 16.67 |
| Female | 38.61 | 27.63 | 58.49 | 73.81 | 70.37 | 83.33 |
| Other | 1.16 | - | - | - | - |  |
| ***Region (%)*** |  |  |  |  |  |  |
| North | 1.93 | 1.87 | 0.00 | 2.38 | 0.00 | 0.00 |
| Northwest | 4.25 | 36.07 | 9.43 | 26.19 | 0.00 | 0.00 |
| Central-West | 7.34 | 5.39 | 3.77 | 14.29 | 0.00 | 0.00 |
| Southwest | 72.97 | 48.95 | 73.58 | 50.00 | 100.00 | 100.00 |
| South | 13.51 | 7.03 | 13.21 | 7.14 | 0.00 | 0.00 |
| ***Exposed to someone with COVID-19 (%)*** |  |  |  |  |  |  |
| Yes | 39.96 | 75.88 | 32.08 | 69.05 | - | - |
| No | 60.04 | 24.12 | 67.92 | 30.95 | - | - |
| ***Have you been diagnosed with COVID-19? (%)*** | |  |  |  |  |  |
| Yes | 4.44 | 25.29 | 1.89 | 16.67 | - | - |
| No | 95.56 | 74.71 | 98.11 | 83.33 | - | - |
| ***Do you have a friend or family member that***  ***died from COVID-19? (%)*** | | |  |  |  |  |
| Yes | 35.52 | 50.82 | 33.96 | 40.48 | - | - |
| No | 64.48 | 49.18 | 66.04 | 59.52 | - | - |
| ***Have you been vaccinated? (%)*** |  |  |  |  |  |  |
| Yes | 0.00 | 94.85 | 0.00 | 92.86 | 0.00 | 100.00 |
| Partially | 0.00 | 3.98 | 0.00 | 7.14 | 0.00 | 0.00 |
| No | 100.00 | 1.17 | 100.00 | 0.00 | 100.00 | 0.00 |
| ***Previous diagnosis of anxiety disorder (%)*** |  |  |  |  |  |  |
| Yes | 33.01 | 34.89 | 37.74 | 40.48 | 14.81 | 95.83 |
| No | 66.99 | 65.11 | 62.26 | 59.52 | 85.19 | 4.17 |
| ***GAD-7*** |  |  |  |  |  |  |
| Mean ± SD | 9.88 ± 6.09 | 9.36 ± 5.64 | 12.89 ± 5.08 | 9.57 ± 5.42 | 14.37 ± 4.31 | 11.29 ± 4.46 |
| Cronbach's alpha | 0.93 | 0.91 | 0.87 | 0.91 | 0.84 | 0.82 |
| ***WSAS*** |  |  |  |  |  |  |
| Mean ± SD | 15.84 ± 10.38 | 8.66 ± 8.33 | 19.28 ± 9.75 | 10.31 ± 7.96 | 21.19 ± 8.88 | 12.08 ± 7.22 |
| Cronbach's alpha | 0.83 | 0.86 | 0.81 | 0.85 | 0.83 | 0.67 |
| ***CAS-BR*** |  |  |  |  |  |  |
| Mean ± SD | 2.64 ± 3.27 | 1.50 ± 2.57 | 2.89 ± 3.01 | 1.31 ± 2.64 | 2.85 ± 3.18 | 1.63 ± 2.06 |
| Cronbach's alpha | 0.84 | 0.85 | 0.79 | 0.88 | 0.82 | 0.76 |

Sociodemographic and psychometric data is divided according to all three groups used in this study. Traces indicates that no data was collected.
